# Expanding Horizons: Robotic Minimally Invasive Direct Coronary Artery Bypass with Left Atrial Appendage Exclusion - A Step Forward in Minimally Invasive Cardiac Surgery

**DOI:** 10.21470/1678-9741-2025-0090

**Published:** 2026-02-13

**Authors:** Abdullah Saad

**Affiliations:** 1 Jinnah Sindh Medical University, Sindh Medical College, Karachi, Pakistan. E-mail: abdullahsaad2005@yahoo.com

Dear Editor,

I read with genuine interest and enthusiasm the innovative work presented by Gregory Fishberger et al.^[1]^ regarding their groundbreaking approach to robotic-assisted minimally invasive direct coronary artery bypass with simultaneous left atrial appendage (LAA) exclusion. This integration of procedures marks a remarkable advancement in minimally invasive cardiac surgery.

The authors' detailed description of their surgical technique deserves particular recognition, especially concerning patient selection methodology and procedural standardization. Nevertheless, several points merit discussion to facilitate broader implementation of this approach^[1,2]^.

Initially, although the authors showcase exceptional outcomes in their carefully curated patient group, additional clarification regarding specific exclusion parameters would benefit institutions planning to adopt this technique. The complexity of mastering such procedures presents unique challenges, and comprehensive guidance on patient selection during early implementation stages would prove invaluable.

The documented procedural durations demonstrate remarkable efficiency, yet insights into their preliminary experience and the volume of cases necessary to achieve such proficiency would be enlightening. This knowledge proves essential for surgical programs contemplating the incorporation of this technique^[1,3]^.

Concerning concurrent LAA exclusion, their approach introduces an innovative solution for stroke risk reduction. Additional information regarding extended follow-up protocols for evaluating LAA closure effectiveness and subsequent modifications in anticoagulation requirements would enhance understanding of long-term outcomes^[1,4]^.

Financial considerations surrounding robotic-assisted procedures remain significant for numerous institutions. While briefly addressed, a comprehensive analysis of cost-effectiveness, particularly regarding reduced hospitalization duration and accelerated recovery periods, would strengthen the rationale for widespread adoption^[1,5]^.

Looking forward, this work creates promising opportunities for expanding minimally invasive cardiac surgery applications. Their success in combining these procedures suggests potential applications for other concurrent cardiac interventions utilizing robotic assistance^[6]^.

I commend the authors for their exceptional contribution to advancing minimally invasive cardiac surgery. Their work exemplifies not only technical excellence but also establishes a framework for future innovations in robotic-assisted cardiac procedures. Their thorough documentation will undoubtedly serve as an invaluable resource for the cardiac surgery community^[6]^.

## Artificial Intelligence Usage

The author declares use of ChatGPT for improving readability. The content produced by the artificial intelligence tool was revised and edited by the author as necessary, and he takes full responsibility for the content to be published.
